# Attitude towards COVID 19 vaccines and vaccine hesitancy in urban and rural communities in Tamil Nadu, India – a community based survey

**DOI:** 10.1186/s12913-021-07037-4

**Published:** 2021-09-21

**Authors:** Kenneth Grace Mascarenhas Danabal, Shiva Shankar Magesh, Siddharth Saravanan, Vijayaprasad Gopichandran

**Affiliations:** 1 Employees State Insurance Corporation Medical College and Post Graduate Institute of Medical Sciences and Research, KK Nagar, Chennai, 600078 India; 2 Department of Community Medicine, Employees State Insurance Corporation Medical College and Post Graduate Institute of Medical Sciences and Research, KK Nagar, Chennai, 600078 India

**Keywords:** COVID 19, Vaccines, Vaccine hesitancy, Attitudes, Cluster analysis

## Abstract

**Background:**

Effective and safe COVID 19 vaccines have been approved for emergency use since the end of 2020 and countries are actively vaccinating their people. Nevertheless, hesitancy towards the vaccines exist globally.

**Objectives:**

We conducted this study to understand the attitudes towards COVID 19 vaccines and hesitancy to accept it among urban and rural communities in Tamil Nadu, India.

**Methods:**

We conducted a community based cross sectional study in urban and rural communities among 564 persons who had not been vaccinated yet, selected through multistage random sampling. The vaccine attitude scale (VAX) was used to measure attitudes towards the vaccines and their acceptance of the vaccine was captured by responses to a direct question.

**Results:**

More than 50% of the respondents had positive attitudes towards the COVID 19 vaccines. Based on their attitudes, they were segmented into four clusters, first with preference for natural immunity compared to vaccines and low concern regarding adverse effects. Second with high level of trust in vaccines and low mistrust. The third cluster members had high level of concern regarding the adverse effects and low levels of mistrust in vaccines and the fourth had high trust in vaccines and low preference for natural immunity. Older individuals with higher education and occupation were more likely to belong to cluster four with high trust in the vaccines. Younger individuals, women, rural residents, belonging to low income labourer class were highly mistrusting of the vaccines. The prevalence of vaccine hesitancy was 40.7% (95% CI – 36.67 - 44.73%), while 19.5% (95% CI = 16.23 - 22.77%) of the respondents were vaccine deniers. While vaccine acceptance was greatest in cluster 1, it was least in cluster 3.

**Conclusions:**

Vaccine hesitancy was high in urban and rural Tamil Nadu. The population could be effectively segmented into groups based on their attitudes and this understanding can be used to develop targeted behaviour change communication campaigns.

## Introduction

The SARS CoV2 pandemic has disrupted the life of people globally [1]. Amidst this grim situation, one of the positive signs of human resilience is the development of effective and safe vaccines, within a year of onset of the pandemic [2]. Vaccines are effective public health tools, which when given to sufficient numbers of people, can halt outbreaks of serious infections [3]. The world currently faces a gross inequity in access to COVID 19 vaccines [4]. While high income countries are making great strides in giving vaccines to all its people, low and middle income countries are still languishing with poor vaccine access [5].

India began the COVID 19 vaccination campaign on 16 January 2021 [6, 7]. As of 04 September 2019, India has vaccinated 67.6 crore people amounting to about 11% of the population fully vaccinated [7]. However, this rate of vaccination is not sufficient to halt the pandemic. There are also substantial inequities in gender, class, and rural-urban divide in coverage of vaccines in India [8, 9]. Currently the main vaccines available in India are Oxford-Astra Zeneca vaccine locally referred to as Covishield, Bharat Biotech-ICMR indigenous vaccine named Covaxin and the Russian Sputnik V vaccine which is imported [6]. In addition several other vaccines have also been given emergency use authorization. While availability and distribution of the vaccines remains a challenge, even in places where vaccines are made available there is vaccine hesitancy [10].

Vaccine hesitancy is the reluctance of people to accept a vaccine that has been proven safe and effective and made available to them for protection against an infectious disease [11]. A 5 C model has been proposed to understand vaccine hesitancy. This model explains that vaccine hesitancy is driven by five main determinants namely: confidence, complacency, convenience, risk calculation and collective responsibility [12]. Vaccine denial and hesitancy has been in existence in populations right from the time of Jenner’s smallpox vaccination. Anti-vaccination groups have always co-existed along with advances in vaccination technology [13]. More recently, the Andrew Wakefield scandal in which the research showed an association between the MMR vaccine and autism created a huge anti-vaccination sentiment and led to reduced vaccine uptake in the United States [14]. In India, vaccine hesitancy and reluctance to accept vaccines exist in significant numbers even for routine immunization. Therefore, vaccine hesitancy associated with COVID 19 vaccines is not new or unexpected.

The overall vaccine hesitancy to routine childhood vaccines in Tamil Nadu showed a rising trend even before the COVID 19 pandemic [15]. There were several instances of hesitancy related to the Measles-Rubella vaccine when it was introduced in campaign mode in the state in 2016 [16]. Consecutive National Family Health Surveys have shown a declining trend of vaccine coverage in Tamil Nadu despite a high performance in all other health care indicators [17]. When the COVID 19 vaccination campaign was launched in India on 16 January 2021, during the early phase there was significant hesitancy among health care providers who were the target population to be covered then [18]. The first wave of COVID 19 had just receded at that time and the second had not yet begun. A popular Tamil film actor-comedian who was an active campaigner for the COVID 19 vaccination drive by the state government, got vaccinated in a public event. The next day on April 17th, he developed a sudden cardiac arrest and died due to causes which were ruled unrelated to the vaccine. But his death created several conspiracy theories about COVID 19 vaccine induced deaths. This further worsened the vaccine hesitancy status in Tamil Nadu [19]. Shortly thereafter, the second wave of COVID 19 struck India and Tamil Nadu was one of the seriously affected states. Hospitals were overcrowded and there was a shortage of oxygen and beds for admitting patients with severe COVID 19. This serious situation created fear among people and substantially increased the demand for vaccination and hesitancy reduced. As of end July 2021, the number of new cases of COVID 19 have reduced in Tamil Nadu and various lock down restrictions have been relaxed. Nevertheless, there is still an anxiety regarding a third wave of infections looming large. It is in this context that there is a need to document and understand the level of vaccine hesitancy in the population. This study was carried out to estimate the level of vaccine hesitancy among people in urban and rural Tamil Nadu and to understand the patterns of hesitancy, degree of hesitation and characteristics of people who hesitate to be vaccinated. This will help design appropriate behaviour change communication campaigns to improve the uptake of the COVID 19 vaccine.

## Methods

### Study setting

We conducted the study in two districts of Tamil Nadu, Chennai and Chengalpattu. Chennai is a coastal metropolitan city and capital of the state of Tamil Nadu with a population of about 7 million. Chengalpattu is a district in northern Tamil Nadu with a population of 2.5 million. As of 22nd July, about 40% of Chennai and 24% of Chengalpattu population were covered by at least 1 dose of COVID 19 vaccine [20].

### Study population

We conducted the study among adult men and women above the age of 18 years who had not received even a single dose of the COVID 19 vaccine. We excluded persons with specific contraindications for COVID 19 vaccine, mental disabilities or persons with communication disabilities whom we could not interview. We calculated the sample size using the formula 4pq/d^2^ to estimate the prevalence of vaccine hesitancy. We assumed the prevalence of vaccine hesitancy to be 42% which was the data obtained from a survey conducted in Tamil Nadu in January 2021 [21]. We used a 95% confidence level, 20% relative precision, design effect of 2 and obtained a sample size of 276. We rounded this off to 280 and sampled 280 participants from urban areas in Chennai and 280 from rural villages in Chengalpattu.

### Sampling method

We adopted a multistage sampling strategy. Chennai is divided into 15 zones, out of which we randomly selected two zones (10% of the zones). In each zone we randomly selected two wards (at least 10% of the wards). From each of these four wards we sampled 70 households by systematic random sampling and randomly sampled an adult member from each household and interviewed them. In Chengalpattu there are 8 panchayat unions, of which we sampled 2 randomly. In these two panchayat unions we sampled 2 villages each. In these four sampled villages we identified 70 households by systematic random sampling and interviewed one adult per household randomly.

### Study instrument

The Vaccine Attitudes Examination (VAX) Scale is a validated instrument to measure vaccine hesitancy [22]. We adapted this scale to the local context and to collect information about attitudes towards COVID 19 vaccination. We translated this instrument to Tamil, the local language. In addition, we also included socio-demographic details of the participants in the questionnaire.

### Data collection

We administered the questionnaire face to face to all the participants and entered their responses into a data collection software application (Google Forms) in our respective mobile phones.

### Statistical analysis

The data were analysed using Statistical Package for Social Sciences SPSS version 25. Initially we counted the responses to all the items in the questionnaire and tabulated them to gain a comprehensive understanding of the data. We entered the Likert type responses (strongly agree – agree – neutral – disagree – strongly disagree) scored from 1 to 5 in an exploratory factor analysis model using Principal Component method of extraction, Varimax rotation and used the eigenvalues greater than 1 to define the number of factors to be extracted. We found the sample to be adequate by the KMO test and model to be fit by Bartlett’s test of sphericity. We computed the regression factor scores. Then we entered the regression factor scores into a hierarchical cluster analysis. Based on the coefficients of the cluster agglomeration schedule, we determined that four clusters could be meaningfully segregated. Then we ran a K means cluster analysis to segment the sample into 4 clusters and identified individual respondents’ cluster membership. We used the cluster centre scores to determine the key attitudinal factors leading to the clustering. This method of segmenting populations for public health interventions using cluster analysis methods has been previously described to be effective [23]. We used Chi Square test to identify the various socio-demographic characteristics that influenced cluster membership. The level of vaccine hesitancy was assessed based on responses to one question “Do you plan to take the COVID 19 vaccine that is currently available in Tamil Nadu?”. The participants responded on a 5 point scale “definitely – probably – not sure – probably not – definitely not”. We categorized ‘probably’, ‘not sure’, ‘probably not’ as vaccine hesitancy and definitely not as vaccine denial. For all the analysis we assumed that *p* value of less than 0.05 indicated statistical significance.

### Ethical considerations

The study was reviewed by the Institutional Ethics Committee of the ESIC Medical College and PGIMSR, KK Nagar, Chennai by an expedited review process. The research was conducted in adherence to the National Ethical Guidelines for Biomedical and Health Research involving Human Participants by the Indian Council of Medical Research, 2017 and the Declaration of Helsinki. The proposal was approved with IEC No. IEC/2021/1/20 dated 20.06.2021. Verbal informed consent was obtained from all participants before collection of data. The institutional ethics committee permitted a verbal consent process in order to minimize the exchange of potential fomites in the form of paper and pen and limit the possibility of transmission of COVID 19. COVID 19 precautions including face mask, hand sanitation, physical distancing were practiced during the data collection to safeguard the researchers as well as the respondents. Respondents were provided adequate privacy during the interview and confidentiality of their information was protected.

## Results

We approached a total of 596 potential respondents out of whom 32 refused to participate and we had 564 participants’ data for analysis with a response rate of 94.6%. The common reasons for non-response were lack of time and lack of interest in participating. Three staunch vaccine deniers refused to participate as they did not want to respond to any questions related to the vaccines. About 70% of the participants were below 45 years of age and 63% were women. About 13% had no schooling, 66% had completed some level of schooling and the remaining 21% had studied beyond high school. About 25% were labourers and 30% were skilled workers. Ten percent of the sample had been confirmed to be infected by the SARS CoV2 virus in the past. The other characteristics of the study population are shown in Table 1.

**Table 1:** Characteristics of the study population

S.No	Characteristic	Categories	Number (%)
1	Age	18-45	387 (68.6%)
		46-65	159 (28.2%)
		66-84	18 (3.2%)
2	Sex	Male	209 (37.1%)
		Female	355 (62.9%)
3	Area of Residence	Urban	282 (50%)
		Rural	282 (50%)
4	Religion	Hindu	472 (83.7%)
		Christian	75 (13.3%)
		Muslim and Others^a^	17 (3%)
5	Literacy of Head of Household	No Schooling	75 (13.3%)
		Schooling	374 (66.4%)
		Diploma, Graduate, PG	115 (20.5%)
6	Occupation of Head of Household	Unemployed	107 (19.0%)
		Labourer	141 (25.0%)
		Clerical	60 (10.6%)
		Skilled Worker	167 (29.6%)
		Executive and Business	78 (13.8%)
		Professional	11 (2.0%)
7	Monthly Family Income	< Rs. 6000	104 (18.4%)
		Rs. 6000 – Rs. 17,999	204 (36.2%)
		Rs. 18,000 – Rs. 30,000	101 (17.9%)
		Rs. 30,000 – Rs. 44,999	28 (5.0%)
		Rs. 45,000 – Rs. 59,999	31 (5.5%)
		Rs. 60,000 – Rs. 100,000	29 (5.1%)
		>Rs. 100,000	9 (1.6%)
		Do not wish to say	58 (10.3%)
8	Marital Status	Unmarried	82 (14.5%)
		Married	452 (80.1%)
		Widowed	30 (5.3%)
9	Type of Family	Nuclear	463 (82.1%)
		Joint	101 (17.9%)
10	Childhood vaccination status	Unsure / Incomplete	102 (18,1%)
		Complete	462 (81.9%)
11	Chronic diseases (multiple responses possible, percentages will not add to 100)	Diabetes	70 (12.4%)
		Hypertension	39 (6.9%)
		Diabetes + Hypertension	22 (3.9%)
		Heart Disease	8 (1.4%)
		Asthma / COPD	16 (2.8%)
		Joint Disease	7 (1.2%)
		Seizures and nervous disorders	6 (1.1%)
		Thyroid diseases	14 (2.5%)
		Other chronic diseases	15 (2.7%)
12	Taking regular medications	Yes	173 (30.7%)
		No	388 (68.8%)
		Can’t Say	3 (0.5%)
13	Were you or your family members diagnosed to have COVID 19?	Yes	57 (10.1%)
		No	507 (89.9%)

^a^others – Jainism, Buddhism

Table 2 shows the responses of the participants on a Likert scale to 13 statements that capture the attitudes towards COVID 19 vaccines. Overall more than 50% of the participants seemed to have a positive attitude towards the vaccines.

**Table 2:** Attitude towards COVID 19 and COVID 19 vaccine

S.No	Statement	Strongly Agree	Agree	Neither Agree Nor Disagree	Disagree	Strongly Disagree
1	I believe that COVID-19 is NOT a real disease	29 (5.1%)	48 (8.5%)	68 (12.1%)	146 (25.9%)	273 (48.4%)
2	I believe that COVID 19 is a new disease and vaccines have not been tested thoroughly	34 (6.0%)	91 (16.1%)	116 (20.6%)	188 (33.3%)	135 (23.9%)
3	I believe that I can feel safe after being vaccinated against COVID 19.	75 (13.3%)	180 (31.9%)	123 (21.8%)	142 (25.2%)	44 (7.8%)
4	I believe that I can rely on vaccines to stop severe COVID 19 disease.	106 (18.8%)	222 (39.4%)	89 (15.8%)	107 (19.0%)	40 (7.1%)
5	I can feel that my family is protected after getting vaccinated against COVID 19	80 (14.2%)	209 (37.1%)	105 (18.6%)	134 (23.8%)	36 (6.4%)
6	I believe that although most COVID 19 vaccines are safe, sometimes there may be problems.	100 (17.7%)	230 (40.8%)	114 (20.2%)	106 (18.8%)	14 (2.5%)
7	I believe that COVID 19 vaccines can cause serious problems in children	66 (11.7%)	125 (22.2%)	206 (36.5%)	139 (24.6%)	28 (5.0%)
8	I worry about serious unknown long-term effects of the COVID-19 vaccine in the future	53 (9.4%)	165 (29.3%))	189 (33.5%)	123 (21.8%)	34 (6.0%)
9	I believe that COVID-19 vaccines make a lot of money for pharmaceutical companies	120 (21.3%)	160 (28.4%)	98 (17.4%)	95 (16.8%)	91 (16.1%)
10	I believe that authorities promote COVID-19 vaccine for political gain and financial gain, not for people’s health	47 (8.3%)	81 (14.4%)	110 (19.5%)	184 (32.6%)	142 (25.2%)
11	I believe that COVID-19 vaccination programs are a big con	35 (6.2%)	63 (11.2%)	111 (19.7%)	232 (41.1%)	123 (21.8%)
12	I believe that Natural immunity lasts longer than vaccination	137 (24.3%)	219 (38.8%)	103 (18.3%)	71 (12.6%)	34 (6.0%)
13	I believe that Natural exposure to germs and viruses gives the safest protection	119 (21.1%)	227 (40.2%)	88 (15.6%)	89 (15.8%)	41 (7.3%)

Table 3 shows the output of the exploratory factor analysis of the 13 item attitude scale. It is seen that the items are grouped into four major dimensions namely; (1) mistrust in the health system and COVID 19 vaccines, (2) trust in effectiveness of the COVID 19 vaccines, (3) concerns regarding adverse effects of the vaccine and (4) preference for natural immunity compared to vaccines. The factor loadings of each item in the four domains are shown in Table 3.

**Table 3:** Dimensions of attitudes towards COVID 19 vaccines

Items Related to attitudes towards COVID 19 vaccine	Dimensions			
	Mistrust in health system and COVID 19 vaccine	Trust in effectiveness of COVID 19 vaccine	Concerns regarding adverse effects of COVID 19 vaccines	Preference for Natural Immunity compared to Vaccines
I believe that authorities promote COVID-19 vaccine for political gain and financial gain, not for people’s health	.753			
I believe that COVID-19 vaccination programs are a big con	.721			
I believe that COVID-19 is NOT a real disease.	.672			
I believe that COVID-19 vaccines make a lot of money for pharmaceutical companies	.623			
I believe that COVID-19 is a new disease, and vaccines have not been thoroughly tested	.526			
I can feel that my family is protected after getting vaccinated for COVID-19		.867		
I believe that I can feel safe after being vaccinated for COVID-19		.837		
I believe that I can rely on vaccines to stop serious COVID-19 disease		.795		
I believe that although most COVID-19 vaccines are safe, sometimes there may be problems			.779	
I worry about serious unknown long-term effects of the COVID-19 vaccine in the future			.712	
I believe that COVID-19 vaccines can cause serious problems in children			.697	
I believe that Natural immunity lasts longer than vaccination				.863
I believe that Natural exposure to germs and viruses gives the safest protection				.828

Factor analysis - extraction by principal component method and varimax rotation

The results of the cluster analysis of the four regression factor scores are shown in Table 4. The factors with the greatest and least numerical scores in that cluster were considered to determine the main attitude of that cluster towards COVID 19 vaccines. The dominant attitude of members of cluster 1, was preference for natural immunity to vaccination (factor score = 1.60132), cluster 2, mistrust in the health system and vaccines (factor score = 0.70998), cluster 3 concern about the adverse effects of vaccines (factor score = 0.09123) and cluster 4 trust in the effectiveness of vaccines (factor score = 0.63677). Members of cluster 1 had the least concern regarding adverse effects of vaccines (factor score = − 0.29324), cluster 2 had least trust in the effectiveness of vaccines (factor score = − 0.79654), cluster 3 had least mistrust in the health system and COVID 19 vaccines (factor score = − 1.26083) and cluster 4 had least preference for natural immunity and rather preferred vaccines (factor score = − 0.69946).

**Table 4:** Classification of the respondents based on their attitudes towards COVID 19 vaccine

	Clusters of respondents based on their attitudes towards COVID 19 vaccine			
	1 (*n* = 82)	2 (*n* = 159)	3 (*n* = 139)	4 (*n* = 184)
Mistrust in health system and COVID 19 vaccine	.35085	.70998	−1.26083	.18260
Trust in effectiveness of COVID 19 vaccine	.59292	−.79654	−.28155	.63677
Concerns regarding adverse effects of COVID 19 vaccines	−.29324	.43559	.09123	−.31464
Preference for Natural Immunity compared to Vaccines	1.60132	−.05033	.03880	−.69946

K means cluster analysis

Table 5 shows the characteristics of members belonging to each of these four clusters.

**Table 5:** Characteristics of the clusters of respondents

S.No	Characteristic	Categories	Cluster 1	Cluster 2	Cluster 3	Cluster 4	*p* value
		18 - 45 yrs	63 (16.2%)	114 (29.4%)	95 (24.5%)	115 (29.7%)	0.005^*^
		46 - 65 yrs	18 (11.3%)	42 (26.4%)	41 (25.8%)	58 (36.5%)	
		> 65 yrs	1 (5.6%)	3 (16.7%)	3 (16.7%)	11 (61.1%)	
2	Sex	Male	28 (13.4%)	46 (22%)	53 (25.4%)	82 (39.2%)	0.025*
		Female	54 (15.2%)	113 (31.8%)	86 (24.2%)	102 (28.7%)	
3	Area	Urban	45 (16%)	44 (15.6%)	69 (24.5%)	124 (44%)	< 0.001*
		Rural	37 (13.1%)	115 (40.8%)	70 (24.8%)	60 (21.3%)	
4	Education	No Schooling	8 (10.7%)	19 (25.3%)	28 (37.3%)	20 (29.7%)	0.003*
		Schooling	60 (16%)	114 (30.5%)	89 (23.8%)	111 (27.7%)	
		Diploma / Graduate / PG	14 (12.1%)	26 (22.6%)	22 (19.1%)	53 (46%)	
5	Occupation	Unemployed	23 (21.5%)	19 (17.8%)	23 (21.5%)	42 (39.3%)	0.002*
		Labourer	13 (9.2%)	59 (41.8%)	39 (27.7%)	30 (21.3%)	
		Clerical	7 (11.7%)	18 (30%)	14 (23.3%)	21 (35%)	
		Skilled Worker	26 (15.6%)	40 (24%)	44 (26.3%)	57 (34.1%)	
		Business and Executive	12 (15.4%)	21 (26.9%)	14 (17.9%)	31 (39.7%)	
		Professional	1 (9.1%)	2 (18.2%)	5 (45.5%)	3 (27.3%)	
6	Monthly Family Income	< Rs. 6000	14 (13.5%)	44 (42.3%)	35 (33.7%)	11 (10.6%)	< 0.001*
		Rs. 6000 - 17,999	33 (16.2%)	68 (33.3%)	52 (25.5%)	51 (25%)	
		Rs. 18,000 - 30,000	16 (15.8%)	15 (14.9%)	24 (23.8%)	46 (45.5%)	
		Rs. 30,000 - 44,999	5 (17.9%)	5 (17.9%)	9 (32.1%)	9 (32.1%)	
		Rs. 45,000 - 59,999	3 (9.7%)	4 (12.9%)	5 (16.1%)	19 (61.3%)	
		Rs. 60,000 - 100,000	5 (17.2%)	10 (34.5%)	4 (13.8%)	10 (34.5%)	
		> Rs. 100,000	1 (11.1%)	2 (22.2%)	0	6 (66.7%)	
7	Did you or your family members have COVID 19?	Yes	9 (15.8%)	9 (15.8%)	8 (14%)	31 (54.4%)	0.001*
		No	73 (14.4%)	150 (29.6%)	131 (25.8%)	153 (30.2%)	

*p<0.05 statistically significant

**Fig. 1:**
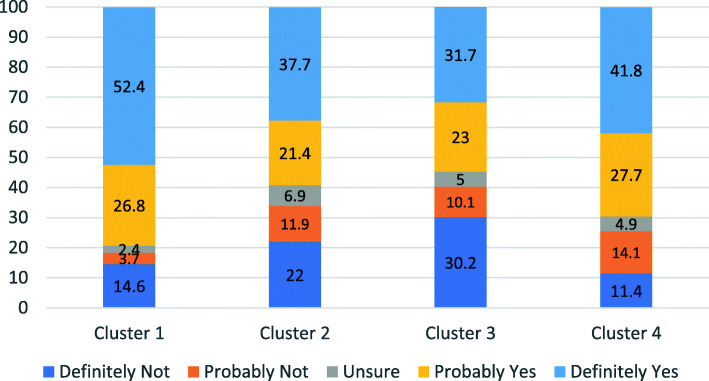
Vaccine hesitancy among different clusters based on attitude towards vaccines

The overall hesitancy towards the vaccines was 40.7% (95% CI – 36.67 - 44.73%), while 19.5% (95% CI = 16.23 - 22.77%) of the respondents were vaccine deniers. The level of vaccine hesitancy among members of the four clusters is shown in Fig. 1.

## Discussion

In this community based survey of attitudes towards COVID 19 vaccines and vaccine hesitancy in urban and rural Tamil Nadu, we found that about half the population had a positive attitude towards the vaccines. About 19.5% of the respondents denied vaccines, whereas 40.7% were hesitant. The main dimensions of the attitudes towards the COVID 19 vaccines were trust in effectiveness of the vaccines, mistrust in the health system and the vaccines, concern regarding adverse reactions of the vaccines and preference for natural immunity compared to the vaccines. Based on these four dominant attitudes the sample was classified into four clusters each of which had a unique characteristic attitude towards the vaccines and different levels of vaccine acceptance. In the following paragraphs we will discuss these findings in detail and their implications for improving vaccine acceptance in Tamil Nadu.

### Preference for natural immunity, not vaccines and low concern for adverse effects

There were 82 (14.5%) members in the cluster with an attitude supporting natural immunity rather than vaccines, and they were also those with least concern regarding adverse effects of the vaccines. People in the age group above 45 years were less likely to belong to this cluster. This could partly be attributed to the fact that the vaccines were offered for persons above 45 years of age as a priority and this age group had greater opportunities to have this vaccine. Also people in older age groups are more susceptible to serious COVID 19 infections and death and therefore the fear of the disease is more in this group leading to a favourable attitude towards COVID 19 vaccines. Unemployed individuals tend to belong in this cluster probably because they were not worried about adverse effects preventing them from going to work. Further we also observed that the highest level of vaccine acceptance was in this cluster (52.4%). Only 3.7% responded that they may probably not get vaccinated and only 2.4% were unsure. Individuals in this cluster need the least behaviour change intervention. Increased awareness generation about the vaccines and reassurance that vaccine mediated immunity is safer than natural exposure to the infection, especially when the infection has dangerous consequences, is required. This would be sufficient to nudge them towards vaccine acceptance.

### Mistrust in the health system, COVID 19 vaccine and least trust in effectiveness of the vaccines

The 159 (28.2%) members in this cluster would be the focus of heightened intervention to create greater awareness and remove misinformation. People in this cluster were typically young 26-45 year of age, women, from rural areas, at lower levels of educational attainment, with low monthly family income and mainly belonging to the labourer class. Despite the high level of mistrust in the vaccine, vaccine deniers were only 22%. Though 40.2% were hesitant, about half of them tend towards accepting the vaccine. Though the mistrusting attitude has led to a 22% denial rate, it is not as high as the denial rate in cluster 3. Even the hesitancy rate is less than the hesitancy in cluster 4. Therefore, there are factors other than these attitudes which govern vaccine hesitancy in this cluster. Behaviour change interventions in this cluster must focus on trust building through active community engagement and transparent dissemination of vaccine related information [24]. It must also focus on creating enabling environments for this vulnerable cluster to access the vaccines and provide physical, informational and emotional support during and after the vaccination [25].

### Concerns regarding adverse effects of the vaccine and low levels of mistrust

This cluster had 139 (24.6%) members, who had low levels of mistrust in the health system and the COVID 19 vaccine, but also had a high level of concern regarding adverse effects of the vaccines. Though they trusted the vaccine, they were concerned about the adverse effects. Young people between 18 and 25 years, professionals, and people with no exposure to COVID 19 infection were more likely to belong to this cluster. It is interesting to note that this cluster had a high proportion of vaccine deniers, probably driven by low age profile and less risk perception and concern regarding adverse effects, despite a high trust in the vaccines. The rates of vaccine hesitancy are comparable with cluster 2 and 4. Interventions directed at this cluster must focus on allaying anxieties regarding the adverse effects, working with employers and encouraging them to provide paid leave for vaccination and in case of any adverse reactions after vaccination, providing incentives for taking vaccines and increasing a sense of solidarity towards the larger society to contribute towards herd immunity.

### Trust in effectiveness of the vaccine and least preference for natural immunity

A total of 184 (32.6%) participants were classified in this cluster. They believed in the effectiveness of vaccines and did not prefer natural immunity. People above 45 years of age, males, urban residents, with higher education, persons employed as clerks, skilled workers and business people and executives, with higher income, and with exposure to COVID 19 infection were more likely to belong to this cluster. This cluster was the opposite of cluster 2. This cluster had the lowest proportion of vaccine deniers. Though the hesitancy rates were similar to that in cluster 2 and 3, they tend towards acceptance. Interventions to create enabling environments to make vaccines accessible and providing support during the process may be important even in this cluster with a strong positive attitude towards the COVID 19 vaccines.

Various studies have closely examined the level of vaccine hesitancy in different parts of the world. The COVID 19 vaccine hesitancy in the UK was reported to be around 35% [26]. A systematic review of COVID 19 vaccine acceptance rates in different parts of the world revealed high acceptance in Malaysia, Indonesia and China and very poor acceptance rates in Italy, Russia, United States and France [27]. A multi country survey conducted in June 2020 before the availability of the vaccine showed that there was wide heterogeneity of factors in various countries that determined vaccine acceptance. Whereas elderly were more likely to accept the vaccine in France and Germany, the young were more likely to accept it in China. High education was associated with greater acceptance in France, Germany and India, whereas low education was more likely to lead to vaccine acceptance in Canada, Spain and UK [28]. In a nationwide survey conducted among medical students in India, vaccine hesitancy was found among 10.6% of the students [29]. This is high given the fact that their level of awareness and risk perception are likely to be high. The major factors driving vaccine hesitancy are false information about the vaccine, lack of sufficient credible information, lack of trust in the health system, and religious factors that deter one from accepting the vaccine [30]. In this study we found similar rates of high vaccine hesitancy in Tamil Nadu. The attitudinal factors driving vaccine hesitancy were also similar.

The strength of our study is that to our best knowledge this is the first attempt at systematically segmenting the population using robust statistical methods based on their attitudes towards the COVID 19 vaccine and understanding the levels of hesitancy related to their attitudes. The understanding that this study provides regarding the subgroups of the population which have high levels of hesitancy and those that have high levels of vaccine denial will guide development of specific targeted interventions to reduce the hesitancy. The findings of this study are directly actionable and can help develop effective behaviour change communication campaigns.

There are some limitations of this study. Firstly, the study is limited to a particular geographic location in Tamil Nadu and is not a state-wide representative survey. We also observed that even among those who had accepted the vaccine, there was substantial hesitation and doubt, but they got vaccinated under pressure by their employers. This aspect of vaccine hesitancy could not be captured. Therefore it is likely that the level of hesitancy that we have found is an underestimate. There is a possibility of socially desirable response bias, as the interviewers identified as being affiliated to the health system in Tamil Nadu. Despite these limitations, the findings of this study are useful because we have demonstrated the effectiveness of the tool and the methods in conducting such a survey to strategically segment the population for public health intervention.

## Conclusion

There is a high level of COVID 19 vaccine hesitancy in Tamil Nadu. This hesitancy is driven by the people’s attitude towards the health system and the vaccine. Apart from these attitudes, social, health system and accessibility factors also probably play a major role in vaccine hesitancy. This study provides a sound understanding of people’s attitudes towards the COVID 19 vaccines and their association with hesitancy and the findings will help design effective behaviour change communication campaigns.

## Data Availability

The datasets used and/or analysed during the current study available from the corresponding author on reasonable request.
